# Pathways to child and adolescent psychiatric clinics: a multilevel study of the significance of ethnicity and neighbourhood social characteristics on source of referral

**DOI:** 10.1186/1753-2000-5-6

**Published:** 2011-03-07

**Authors:** Anna-Karin Ivert, Robert Svensson, Hans Adler, Sten Levander, Per-Anders Rydelius, Marie Torstensson Levander

**Affiliations:** 1 Faculty of Health and Society, Malmö University, SE-205 06 Malmö, Sweden; 2 Department of Woman and Child Health, Karolinska Institutet, Astrid Lindgren Children's Hospital, Karolinska University Hospital, SE-171 76 Stockholm Sweden; 3 Department of Medical sciences, Malmö, Lund University, SE-221 84 Lund, Sweden

## Abstract

**Background:**

In the Swedish society, as in many other societies, many children and adolescents with mental health problems do not receive the help they need. As the Swedish society becomes increasingly multicultural, and as ethnic and economic residential segregation become more pronounced, this study utilises ethnicity and neighbourhood context to examine referral pathways to child and adolescent psychiatric (CAP) clinics.

**Methods:**

The analysis examines four different sources of referrals: family referrals, social/legal agency referrals, school referrals and health/mental health referrals. The referrals of 2054 children aged 11-19 from the Stockholm Child-Psychiatric Database were studied using multilevel logistic regression analyses.

**Results:**

Results indicate that ethnicity played an important role in how children and adolescents were referred to CAP-clinics. Family referrals were more common among children and adolescents with a Swedish background than among those with an immigrant background. Referrals by social/legal agencies were more common among children and adolescents with African and Asian backgrounds. Children with Asian or South American backgrounds were more likely to have been referred by schools or by the health/mental health care sector. A significant neighbourhood effect was found in relation to family referrals. Children and adolescents from neighbourhoods with low levels of socioeconomic deprivation were more likely to be referred to CAP-clinics by their families in comparison to children from other neighbourhoods. Such differences were not found in relation in relation to the other sources of referral.

**Conclusions:**

This article reports findings that can be an important first step toward increasing knowledge on reasons behind differential referral rates and uptake of psychiatric care in an ethnically diverse Swedish sample. These findings have implications for the design and evaluation of community mental health outreach programs and should be considered when developing measures and strategies intended to reach and help children with mental health problems. This might involve providing information about the availability and accessibility of health care for children and adolescents with mental health problems to families in certain neighbourhoods and with different ethnic backgrounds.

## Background

Since the late 1920s and using the Health Registers in Sweden, cohorts of child and adolescent psychiatric (CAP) patients have been described and followed over different periods of time up to 30 years[1-4]. These studies have given information regarding the characteristics of the children, adolescents and their families seeking help from child and adolescent psychiatric services. The results have raised questions about different possibilities for anticipating who these children are prior to becoming patients in order to discuss preventive measures. Recent results [4] indicate paths into later CAP-care and care in General Psychiatry which can be identified among patients in paediatric health care.

As the Swedish society, like that of most other Western European countries, becomes increasingly multicultural, and as residential segregation, economic as well as ethnic, becomes more pronounced, the challenge of meeting children's and adolescents' mental health needs requires us to focus more attention on the issues of ethnicity and residential neighbourhood. The Swedish population has changed over the last decades from a relatively homogenous group to a population where almost 20 percent of all children and adolescents under age 18 are either born abroad or born in Sweden with two parents born outside Sweden. The Swedish immigrant population is primarily comprised of three groups; labour immigrants who arrived from the other Nordic countries and southern Europe during the 1950s and 1960s; political refugees from Latin America (mainly from Chile) and Iran who arrived during the 1980s; and refugees who arrived during the final decade of the 20^th ^and the first years of the 21^st ^century from the former Yugoslavia, the former Soviet union, Iraq and Somalia [5]. According to data from the Swedish National Board of Health and Welfare, immigrants from non-European countries had worse health [5], were more likely to have low socioeconomic status, and more often lived in disadvantaged neighbourhoods [6].

A number of European and North American studies have found differences in children's mental health and mental healthcare utilization to be associated with both ethnicity and characteristics of the neighbourhood of residence [7-11]. Less is known about how ethnicity and neighbourhood characteristics affect the way children and adolescents come into contact with mental healthcare services.

Many children and adolescents with mental health problems do not receive the help they need [12,13]. An important first step towards providing appropriate prevention and care is extended knowledge on how children and adolescents with mental health problems are referred to psychiatric care. Parents perceiving that their child has mental health problems is often a prerequisite for a referral to mental health care, and parental awareness of the existence of a problem has been identified as the key initial step in help-seeking [14]. However, referrals to mental health care may also be made by other adults, such as representatives of social agencies or school personnel. According to Verhulst [15], the recognition of children's behaviour as being problematic by parents or other adults is dependent on the latter's "awareness of the problem, their distress threshold, their educational level, beliefs, and attitudes, as well as other cultural and environmental factors" [15]. Together these factors will affect which children will be referred to psychiatric care and by whom. The present study, using Swedish health and population registers, focuses on those children and adolescents who have already entered the psychiatric care system and provides insights into the characteristics of children and adolescents who are in treatment by assessing the question of whether children's and adolescents' referral pathways to child and adolescent psychiatric clinics in a Swedish sample vary by ethnicity and neighbourhood of residence.

### The role of ethnicity in children's referrals to mental healthcare

Previous research indicates that ethnicity may be associated with how individuals come into contact with mental healthcare services [16-20]. For example, African American children tend to be referred to mental healthcare services by social agencies, child welfare and the juvenile justice system more often than children of Caucasian origin [17], while children of Latino origin are more often referred by the school system [21] or their families [17]. Furthermore, a study from the United Kingdom showed that family referrals were rare among Bangladeshi children compared to children with other ethnic backgrounds [22], and results from an Irish study indicates that immigrant children more often were referred through schools than were children with Irish background [23].

There is no simple explanation for the observed differences in sources of referrals among children from different ethnic groups. One explanation that is often presented argues that referral patterns are influenced by socio-cultural factors [16,18,24-26]. Children who are recent immigrants may experience problems in adjusting to their new environment, and school staff, for example, may judge their behaviour as deviant and consequently refer them to the mental healthcare [21]. Socio-cultural differences may also manifest themselves in ethnic differences in families' perceptions of whether or not a problem should be defined as mental health-related, and of whether the problem warrants a mental health care referral [16,25-28]. Previous research has found ethnic differences in parental recognition of mental health problems in their children [28,29], indicating differences in tolerance thresholds for mental health problems. Even if the threshold for what is considered a mental health problem would be the same across ethnic groups there may be a reporting bias. A vignette study by Chavez et al [30] indicated that Latino children were judged as less in need of service than children with Anglo names, by parents as well as mental health care providers (se also [31]). The decision to seek help for a mental health problem may be associated with stigmatization [17,18,32,33]. In some ethnic groups, the stigma of having mental illness in the family may prevent parents from referring their children to mental health care. There are also researchers who argue that children from some ethnic groups are more likely to be labelled by social agencies as being in need of mental health care than others [21], and that ethnic differences in referral pathways emerge as a consequence of this. There are also results indicating the presence of ethnic differences in the type of problems that are recognised and referred, with internalising problems being more likely to remain unreferred among minority adolescents than among Caucasian adolescents, whereas African American adolescents were more likely to be referred for externalising behaviour [34].

A majority of the previous research on ethnic differences in children's referral pathways has been conducted in the USA, and the results may not be directly applicable to a Swedish and/or Western European context. The social structure and the ethnic composition of Sweden, with her relatively new immigrant population, differ significantly from those of the USA. Nevertheless similar patterns of discrimination and of inequalities in access to health care may be present in the Swedish context and affect referral patterns to Child and adolescent psychiatric care.

### The role of neighbourhood characteristics in children's referrals to mental health care

There is a growing body of research examining the association between neighbourhood characteristics and mental health problems among children [7,8,10,11,35,36]. Factors such as neighbourhood socioeconomic deprivation and, social capital (often measured as social cohesion and informal social control) have been identified as having significant effects on children's mental health over and above individual level variables [7,36-38]. Socioeconomic deprivation and social capital have been hypothesised to affect mental health in children (and adults) through factors such as access to family advice and support, informal social networks with neighbours that might contribute to support, child rearing methods, perceptions of risk and danger, and access to resources in the community (see for example [9,11,35,39]. These same factors may also affect the ways in which children and adolescents are referred to psychiatric care. Previous studies have found that individuals in poor communities have less access to speciality care [40], and neighbourhood poverty has been identified as key to understand ethnic disparities in mental health care utilisation [41]. In a recent study Carson, Cook and Alegria [42] found that Haitian youth living in high-poverty areas were less likely to receive adequate mental health care compared to Haitian youths living in low-poverty areas.

The availability of health care options in the neighbourhood, and of knowledge on how to access them, may influence how individuals experience and come in contact with mental health care services [18]. Social norms relating to which behaviours are viewed as undesirable and deviant may also influence referral patterns to mental health care, i.e. the behaviours that are viewed as acceptable and normal may vary by neighbourhood context [16], just as child rearing methods and support from informal social networks. The availability of and knowledge about health care options may, like the propensity to seek help, be correlated with neighbourhood levels of socioeconomic deprivation and social capital. A theoretical model developed by Wikström [43] to explain another kind of problem, i.e. crime, suggests that community structure (resources and rules) influences both the personalities and lifestyles of the individuals who live there, and also their routine behaviours. This implies that the characteristics of the neighbourhood of residence may influence how people define health and ill-health, and may consequently affect the type of problems for which they choose to contact mental health services, for example.

A family referral may be interpreted as indicating the parents' recognition and acceptance of the child's problem and of the fact that the problem warrants mental health care, and also that the parents believe that mental health care services can be helpful in solving the problem. Referrals by an external agency, on the other hand, may be associated with a higher level of coercion, and even if where the approval of the parents is required, the parents' support for and confidence in the care provided may not be as strong as if the parents had themselves initiated the referral. Parental recognition of their child's mental health problems may also imply early detection and treatment of the problem. A better understanding of the ways in which ethnicity and residential neighbourhood influence children's referral patterns to mental health care services may provide important insights into how best to design and develop health promotion strategies to reach children with mental health problems. The key issue for this paper was to study the referral sources by which children and adolescents are referred to CAP-clinics (i.e. who initiates the contact with mental health care services), and whether referral pathways differ by ethnicity and neighbourhood of residence. In the analysis we examine four different referral sources: family referrals, social/legal agency referrals, school referrals and health/mental health referrals. We hypothesise that children will be referred by different sources depending on (1) ethnic background and (2) the neighbourhood of residence and its level of neighbourhood socioeconomic deprivation.

## Methods

### Study population and data

The Stockholm Child-Psychiatric Database comprises approximately 7600 children who have been in contact with child and adolescent psychiatric clinics (CAP-clinics) in the county of Stockholm. The CAP-system comprises the county of Stockholm's outpatient child psychiatric guidance clinics for children and adolescents up to the age of 20. These clinics maintain a computerised system for patient statistics based on structured information that is gathered in relation to each child who attends a CAP-clinic. The clinician (child psychiatrist, psychologist or social worker) is required to fill out a form with information on variables such as cause of referral, diagnoses (according to the DSM-IV, diagnoses are primarily filled out for those children who at some time have subjects for inpatient care), psychosocial stressors, length and type of treatment, referral source, residential neighbourhood, and social background. The Stockholm Child-Psychiatric Database includes children born in 1989 or earlier who had contacts with CAP-clinics that were concluded between 2003 and 2005.

The present study includes only those children who had their first contact with the CAP-clinics in the year 2000 or later, and who were living in the municipality of Stockholm at the time their contacts with the CAP-clinics were concluded.

The Stockholm municipality is divided into 132 neighbourhoods. In this study, a neighbourhood is synonymous with a census tract. The child/adolescent's residential neighbourhood is measured at the time of their final appointment. The present study only includes neighbourhoods from which there are at least 10 children in the Stockholm Child-Psychiatric Database (see appendix for a discussion on number of children per neighbourhood). This yields a final sample of 2054 children and adolescents (representing about 94 percent of the children who attended child and adolescent psychiatric clinics in the Stockholm municipality) from 82 neighbourhoods (with a range of 10-74 children per neighbourhood).

### Measures

#### Referral source

The dependent variable analysed in this study is the referral source that initiated the child's or adolescent's contact with the CAP-clinic. Referral sources were grouped into four categories; family referrals (i.e. family members and self-referrals; n = 1662; 80.9%), social/legal agency referrals (i.e. social services, lawyers; n = 162; 7.9%), school referrals (i.e. teachers, school health care staff, after school centres; n = 414; 20.2%), and health/mental health referrals (i.e. general practitioner, child health centre, adult psychiatric services; n = 722; 35.2%).The variables are dichotomized as 1 = the child or adolescent has been referred to a CAP-clinic by the source at least once, and 0 = the child has never been referred to a CAP-clinic by the source. As a result of data constraints it is not possible to say anything about which source referred the child/adolescent in connection with their first contact with the child and adolescent psychiatric clinics, but rather only whether or not the child/adolescent has been referred by a particular source at any time.

*Ethnicity *was measured on the basis of the parents' country of birth; children whose parents were both born abroad are considered as having an immigrant background. The children were classified into one of six ethnic groups: Swedish, Nordic (other than Sweden), European, Asian, South American, and African. All these subgroups obviously contain important within-group heterogeneity. However it is not possible to create smaller, more homogenous groups since for some children and adolescents, the available data refer only to the region of origin (e.g. other Asian). In the analysis, the ethnicity measure is employed in the form of five dummy variables for Nordic, European, Asian, South American and African background, with Swedish background being used as the reference category. Initial analysis did not show any significant differences in referral source between first and second generation immigrants and therefore we did not distinguish between first and second generation immigrants in the analysis.

Three demographic variables that may be associated with referral patterns to CAP-clinics were included in the analysis as control variables; gender, age, and family structure. *Age *at first contact was included in the analysis as a continuous variable. *Family structure *was divided into two categories based on whether or not the child was living with both parents.

#### Neighbourhood socioeconomic deprivation

The neighbourhood level variable used in this study is neighbourhood socioeconomic deprivation, and can be described as representing socioeconomic status at the neighbourhood level. In previous studies, socioeconomic deprivation has been found to be associated with differences in children's mental health [7,34] and we wanted to examine if deprivation was also associated with children's referrals to mental health care services. Data on Neighbourhood deprivation are derived from the City of Stockholm statistics department (USK), and refer to the year 2004. At the neighbourhood level, four variables are used to measure the level of socioeconomic deprivation in each area: the proportion unemployed, the proportion with less than 12 years of education, the proportion of low income earners (persons with an income below 120,000 SEK/year), and the proportion of high income earners (persons with an income above 360,000 SEK/year). In order to summarise these data to a single construct, a factor analysis was carried out. All four variables loaded highly on a single factor using non-rotated Principal Axis Factoring (loadings >.76), which explained 78 percent of the total variance. Regression factor scores were calculated for the socioeconomic deprivation construct, yielding a continuous, normal distributed variable, with a mean value of 0. The socioeconomic deprivation variable is included in the analysis as a continuous variable; a high value indicates a high level of neighbourhood socioeconomic deprivation.

### Analytical approach

In order to test our hypothesis, a number of multilevel (hierarchical) logistic regressions were carried out using HLM 6.6 [44]. The multilevel approach allows us to examine neighbourhood effects and individual level effects in the same model, and enables us to determine whether neighbourhood socioeconomic deprivation affects children's and adolescent's pathways to care over and above the effects of individual characteristics.

The Intra Class Correlation (ICC) has been calculated in order to estimate the between-neighbourhood variance. According to Snijders & Bosker [45] the ICC in multilevel logistic regression is calculated as: Neighbourhood variance/(neighbourhood variance + π^2^/3). The larger the ICC is the more of the variance in the outcome variable, i.e. source of referral, can be attributed to characteristics in the neighbourhood where the child/adolescent lives. The odds ratios (ORs) in multilevel logistic regression models are interpreted in the same way as the estimates in a single-level logistic regression.

We estimated four models for each source of referral, with children/adolescents at the first level, and neighbourhoods at the second level. Model I represents what is referred to as an empty model, which is an intercept only model with no independent variables. The empty model indicates whether there are any significant differences between neighbourhoods, and also shows the way the variance is distributed between individuals and neighbourhoods. In Model II, ethnicity was added in order to test its correlation with the dependent variable, and to establish whether the neighbourhood variation remains significant after controlling for compositional effects associated with ethnicity. Model III included the control variables gender, age and family structure in order to examine their effect on the correlation between ethnicity and source of referral and on the between-neighbourhood variance. In the final model (model IV), neighbourhood socioeconomic deprivation was introduced in order to test whether the level of neighbourhood socioeconomic deprivation had an independent effect on the source of referral.

### Ethics

The study was approved by the Ethics Committee at Karolinska Institutet, Stockholm (Regionala etikprövningsnämnden, Stockholm).

## Results

Table 1 provides information on the distribution of individual- and neighbourhood-level variables by ethnicity. Approximately 18 per cent of the children in the final sample have an immigrant background. Almost 70 percent of the children with an immigrant background came from countries outside Europe (Asia 40%, South America 17% and Africa 11%). A majority of the children and adolescents in the sample are girls, and did not live with both of their parents. The average age at first contact is 15 years (range 11 to 19) for both immigrant children and children with a Swedish background, with the exception of the Asian and African groups where the average age at first contact is 16 years. Living in a neighbourhood with high levels of socioeconomic deprivation was more common among children with immigrant background, especially among children from the Asian and African group.

**Table 1:** Characteristics of the 2054 children in the sample (percentages/mean)

Variable	Sweden (n = 1678, 82%)	Nordic countries (other than Sweden) (n = 54, 3%)	Other European countries (n = 73, 4%)	Asia (n = 145, 7%)	South America (n = 64, 3%)	Africa (n = 40, 2%)
*Gender *(%)						
Boys	34	24	43	41	47	48
Girls	66	76	57	59	53	52
						
*Age *(mean)	15	15	15	16	15	16
						
*Family structure *(%)						
Living with both parents	45	35	48	45	34	28
Not living with both parents	55	65	52	55	66	72
						
*Neighbourhood socioeconomic deprivation¹ (%)*						
Low	35.7	20.4	19.2	8.3	15.6	10.0
Middle	36.9	33.3	30.1	22.8	20.3	22.5
High	27.4	46.3	50.7	69.0	64.1	67.5

¹ For descriptive statistics, level of socioeconomic deprivation was divided into three groups (based on tertile cut-off points) ranging from low levels to high levels of socioeconomic deprivation

The most common source of referral was family/self referrals. However a chi-square test of differences in referral sources indicated that children with Swedish, Nordic or South American backgrounds were more often referred to child and adolescent psychiatric clinics by a family member than were children with a background in European countries (except the Nordic countries), Asia or Africa (*x*^2 ^= 38.97, p < .001). In the African group, just over 50 percent had been referred by a family member, as compared to almost 80 percent in the total sample. Overall, children with an immigrant background were more often referred to CAP-clinics by social services/legal agencies, the school system or health/mental health care services than were children with a Swedish background (*x*^2 ^= 26.49, p < .001; *x*^2 ^= 16.35, p < .01; *x*^2 ^= 17.06, p < .01).

Tables 2, 3, 4 and 5 present odds ratios for each referral source respectively following a stepwise inclusion of individual and neighbourhood variables.

**Table 2:** Odds ratios for family referrals, with 95% confidence interval (CI). N = 2054

	Model I Empty model	Model II	Model III	Model IV
**Fixed effects**				
*Individual-level variables*				
Ethnicity				
Swedish		1 (reference)	1 (reference)	1 (reference)
Nordic countries (other than Sweden)		1.15 (0.56-2.39)	1.05 (0.48-.2.26)	1.23 (0.58-2.57)
Other European countries		0.50 (0.30-0.84)*	0.45 (0.26-0.78)**	0.53 (0.31-0.92)*
Asia		0.67 (0.44-1.02)	0.69 (0.47-1.03)	0.88 (0.59-1.33)
South America		1.06 (0.53-2.11)	0.94 (0.47-1.87)	1.17 (0.59-2.34)
Africa		0.28 (0.14-0.60)**	0.30 (0.14-0.65)**	0.35 (0.17-0.72)**
				
Gender				
Girl			1 (reference)	1 (reference)
Boy			0.91 (0.73-1.14)	0.95 (0.76-1.20)
				
Age			0.78***	0.78***
				
Family structure				
Living with both parents			1 (reference)	1 (reference)
Not living with both parents			0.86 (0.67-1.11)	0.91 (0.71-1.17)
				
*Neighbourhood-level variable*				
Neighbourhood socioeconomic deprivation				0.68 (0.62-0.76)***
**Random effects**				
Between-neighbourhood variance (SE)^1^	0.266 (0.515) ***	0.205 (0.452) ***	0.173 (0.416)***	0.007 (0.084)
**ICC (%)**	7.5	5.9	5.0	0.2
Explained variance (%)	-	21.3	33.3	97.3

***p < .001, **p < .01, *p < .05. ^1 ^Standard error.

**Table 3:** Odds ratios for social/legal agency referrals, with 95% confidence interval (CI). N = 2054

	Model I Empty model	Model II	Model III	Model IV
**Fixed effects**				
*Individual-level variables*				
Ethnicity				
Swedish		1 (reference)	1 (reference)	1 (reference)
Nordic countries (other than Sweden)		1.41 (0.65-3.06)	1.16 (0.51-2.63)	1.04 (0.46-2.36)
Other European countries		2.19 (1.13-4.24)*	2.21 (1.10-4.44)*	1.84 (0.86-3.94)
Asia		1.82 (1.09-3.05)*	2.03 (1.22-3.38)**	1.50 (0.85-2.67)
South America		1.70 (0.77-3.75)	1.32 (0.58-2.97)	1.06 (0.45-2.50)
Africa		4.57 (2.43-8.60)***	4.51 (2.30-8.85)***	3.55 (1.77-7.10)**
				
Gender				
Girl			1 (reference)	1 (reference)
Boy			1.09 (0.76-1.57)	1.08 (0.76-1.54)
				
Age			0.78 (0.71-0.85)***	0.77 (0.71-0.85)***
				
Family structure				
Living with both parents			1 (reference)	1 (reference)
Not living with both parents			3.11 (2.12-4.56)***	2.92 (1.98-4.29)***
				
*Neighbourhood-level variable*				
Neighbourhood socioeconomic deprivation				1.29 (1.10-1.52)**
**Random effects**				
Between neighbourhood variance (SE)^1^	0.088 (0.296)	0.024 (0.154)	0.007 (0.090)	0.008 (0.091)
**ICC (%)**	2.6	0.7	0.2	0.2
Explained variance (%)	-	73.1	92.3	92.3

***p < .001, **p < .01, *p <.05. ^1 ^Standard error.

**Table 4:** Odds ratios for school referrals, with 95% confidence interval (CI). N = 2054

	Model I Empty model	Model II	Model III	Model IV
**Fixed effects**				
*Individual-level variables*				
Ethnicity				
Swedish		1 (reference)	1 (reference)	1 (reference)
Nordic countries (other than Sweden)		1.51 (0.88-2.58)	1.54 (0.87-2.72)	1.46 (0.80-2.64)
Other European countries		1.12 (0.64-1.93)	1.06 (0.62-1.80)	0.99 (0.61-1.60)
Asia		1.57 (1.11-2.22)*	1.66 (1.17-2.34)**	1.48 (1.07-2.05)*
South America		2.08 (1.32-3.28)**	1.94 (1.23-3.07)**	1.79 (1.13-2.85)*
Africa		1.85 (0.88-3.90)	2.00 (0.97-4.15)	1.81 (0.84-3.90)
				
Gender				
Girl			1 (reference)	1 (reference)
Boy			1.45 (1.15-1.83)**	1.45 (1.15-1.82)**
				
Age			0.84 (0.79-0.90)***	0.84 (0.79-0.90)***
				
Family structure				
Living with both parents			1 (reference)	1 (reference)
Not living with both parents			0.76 (0.63-0.93)**	0.74 (0.61-0.90)**
				
*Neighbourhood-level variable*				
Neighbourhood socioeconomic deprivation				1.11 (0.99-1.24)
**Random effects**				
Between-neighbourhood variance (SE)^1^	0.052 (0.228)	0.022 (0.149)	0.015 (0.124)	0.006 (0.080)
**ICC (%)**	1.6	0.7	0.5	0.2
Explained variance (%)	-	56.3	68.8	87.5

***p <.001, **p <.01, *p <.05. ^1 ^Standard error.

**Table 5:** Odds ratios for health/mental healthcare referrals, with 95% confidence interval (CI). N = 2054

	Model I Empty model	Model II	Model III	Model IV
**Fixed effects**				
*Individual-level variables*				
Ethnicity				
Swedish		1 (reference)	1 (reference)	1 (reference)
Nordic countries (other than Sweden)		1.37 (0.75-2.50)	1.41 (0.76-2.62)	1.39 (0.74-2.61)
Other European countries		1.39 (0.86-2.25)	1.34 (0.82-2.20)	1.32 (0.83-2.11)
Asia		1.57 (1.16-2.13)**	1.62 (1.18-2.22)**	1.58 (1.19-2.11)**
South America		2.11 (1.24-3-57)**	2.07 (1.22-3.51)**	2.03 (1.21-3.38)**
Africa		1.07 (0.49-2.34)	1.16 (0.55-2.45)	1.13 (0.52-2.46)
				
Gender				
Girl			1 (reference)	1 (reference)
Boy			1.24 (1.02-1.50)*	1.24 (1.02-1.50)*
				
Age			0.90 (0.85-0.95)***	0.90 (0.85-0.95)***
				
Family structure				
Living with both parents			1 (reference)	1 (reference)
Not living with both parents			0.70 (0.60-0.83)***	0.70 (0.60-0.83)***
				
*Neighbourhood-level variable*				
Neighbourhood socioeconomic deprivation				1.02 (0.92-1.13)
**Random effects**				
Between-neighbourhood variance (SE)^1^	0.027 (0.164)	0.011 (0.105)	0.005 (0.074)	0.006 (0.078)
**ICC (%)**	0.8	0.3	0.2	0.2
Explained variance (%)	-	62.5	75.0	75.0

***p <.001, **p <.01, *p <.05. ^1 ^Standard error.

### Fixed effects

Table 2 shows that the odds for ever having been referred to a CAP-clinic by the family were significantly lower for those children with a background in African countries (OR = 0.28) or in Europe outside the Nordic countries (OR = 0.50). This association remains after controlling for individual- and neighbourhood level variables. In the final model, the odds for ever having been referred to a CAP-clinic by the family were also significantly lower for those children who were older at the time of their first contact with a CAP-clinic (OR = 0.78), and for those children who lived in a neighbourhood with a low level of socioeconomic deprivation (OR = 0.68).

Odds ratios for referrals to child and adolescent psychiatric clinics by social/legal agencies (table 3) were significantly higher for children with a background in Europe outside the Nordic countries (OR = 2.21) or in Asian (OR = 2.03) and African (OR = 4.51) countries; this association remains after adjusting for sex, age, family structure. When neighbourhood socioeconomic deprivation is entered in model IV just African background remain significantly associated with social/legal agency referrals (OR = 3.55). In the final model, referrals by social/legal agencies were also significantly associated with age at first contact (OR = 0.77), not living with both parents (OR = 2.92), and living in a neighbourhood with a high level of socioeconomic deprivation (OR = 1.29).

Tables 4 and 5 show that Children from an Asian or South American background were more often referred to CAP-clinics by schools (OR = 1.57/2.08) and other health/mental health care institutions (OR = 1.57/2.11). This association remains after adjusting for individual- and neighbourhood level variables. In the final models, the odds for being referred to CAP-clinics by schools or other health/mental health care institutions were higher for boys (OR = 1.45/1.24), and for those who did not live with both of their parents (OR = 0.74/0.70). Referrals by schools and other health/mental health care institutions were also significantly associated with age at first contact (OR = 0.84/0.90). There were no significant association between referrals by schools or health/mental health care institutions and level of neighbourhood socioeconomic deprivation.

### Random effects

The only significant neighbourhood effect was found in relation to family referrals (Table 2). The results from the empty model indicate that about 7.5 percent of the variance in family referrals to child and adolescent psychiatric clinics may be explained by the neighbourhood of residence. This neighbourhood effect decreases by approximately 21 percent when ethnicity is adjusted for, indicating that part of the between-neighbourhood variance is due to compositional effects, i.e. people with certain characteristics (e.g. ethnicity) that affect the probability of the family being the source of referral tend to cluster in the same neighbourhoods. Adjustment for the other individual-level variables reduces the neighbourhood effect further. When neighbourhood socioeconomic deprivation is introduced in the final model (model IV), the between-neighbourhood variance in family referrals to CAP-clinics is reduced to insignificance. Individual- and neighbourhood-level variables together explain approximately 97 percent of the initial variation in family referrals between neighbourhoods.

No significant neighbourhood effects on referrals are found in relation to referrals by the social services/legal agencies, the school system, or health/mental health care services (Tables 3, 4 and 5).

## Discussion

This study has addressed the question of how children and adolescents are referred to CAP-clinics, and whether referral patterns vary by ethnicity and neighbourhood of residence. This is an area of research where the knowledge is limited, especially outside the US context, and this study provides important knowledge on factors that influence children's and adolescents' pathways into care, factors which can also be used for developing preventive measures. In line with our first hypothesis, and also with previous research as described in the introduction, the results indicate that ethnicity plays an important role in how children and adolescents are referred to child and adolescent psychiatric clinics. Family referrals were found to be more common among children and adolescents with a Swedish background than they were among those with an immigrant background. Referrals by social/legal agencies were more common among children and adolescents with African and Asian backgrounds and among children and adolescents from Europe outside the Nordic countries. Children with an Asian or South American background were more likely than children and adolescents with other backgrounds to have been referred by schools or by health/mental health care services.

The data available in this study cannot explain the observed differences in the source of referral between ethnic groups. In a review by Morgan et al. [18], however, three areas are identified as being important to an understanding of differences in referrals to mental health care between individuals from different ethnic backgrounds; (1) social networks, (2) cultural contexts and beliefs about mental illness, and (3) the range of available care options. Social networks may be important to an understanding of ethnic disparities in referral patterns in different ways. Some parents might choose to turn to their social network for help instead of turning to the public health care system. The social network could also be an important source of variation in attitudes towards professional help seeking, and in knowledge about available health care options. A study by Lindsey et al [20] found that having a large social network was associated with the utilisation of school mental health services among African American adolescents.

Cultural contexts and beliefs about mental illness may be associated with parental differences in tolerance thresholds and their perceptions of whether or not a problem behaviour is defined as being mental health-related, and parents' attitudes to psychiatric care [16,25,26,28]. An additional explanation may be that the level of stigmatization associated with mental health problems differs between ethnic groups [17,18,32]. It is also possible that parents of Swedish origin have better knowledge of, and access to, available health care options, and that contacting child and adolescent psychiatric clinics is a natural way for them to deal with their children's problems. However, results from a British study showed no significant differences in level of awareness mental health care services between Pakistani and white British mothers, though Pakistani mothers were less likely to consider referral for problems judged as mild or moderate [27].

The results from the present study indicate that agency referrals were more common among children and adolescents with an immigrant background (with the exception of children and adolescents from the Nordic countries). This may be associated with the behaviour of children who do not belong to the majority population being more likely to be labelled as deviant by social agencies, for example, or school staff. But this over-reporting of children with an immigrant background may also be a consequence of these children being less likely to be referred to child and adolescent psychiatric clinics by their families, and of their problem behaviour, as a result, having being identified and reported by an external party. Previous studies have identified school personnel as important actors for both the detection of mental health problems and for making referrals to mental health care [33,46,47]. As has already been noted, Engqvist & Rydelius [4] have found that information from the paediatric care sector plays an important role in identifying and supporting children with mental health problems, since extensive and recurrent hospital care during childhood is associated with an increased likelihood of subsequent psychiatric illness.

The hypothesised relationship between neighbourhood of residence and differences in referral sources was found in this study to be ambiguous. On the one hand we found a significant neighbourhood effect on family referrals, i.e. it was more likely for children and adolescents to be referred to child and adolescent psychiatric clinics by the family in some neighbourhoods than it was in others. On the other hand no significant neighbourhood effect was found in relation to social/legal agency referrals, school referrals, or health/mental health service referrals.

The finding showing that there is a neighbourhood effect on family referrals is in line with the theoretical discussion presented in the introduction, i.e. that characteristics of the social settings in which children and adolescents live will have an independent effect on the sources by which they are referred by to child and adolescent psychiatric clinics. Given the rather strong association found between ethnicity and family referrals, it might be assumed that the neighbourhood effect was largely due to compositional factors, but controlling for ethnicity only reduced the neighbourhood effect by approximately 20 percent. The structure of the community may influence the routine behaviour of the people who live there. Families living in a neighbourhood where referrals to child and adolescent psychiatric clinics are part of the routine will perceive this as a way of dealing with their children's problems to a greater extent. Some of the mechanisms discussed in connection with ethnic differences in referral patterns may also serve as explanations for the observed differences between neighbourhoods. Knowledge about and access to different health care options may differ between neighbourhoods in the same way as the type of behaviours that are regarded as mental health-related and opinions on how to deal with problems of this kind. In the introduction we hypothesised that the neighbourhood level of socioeconomic deprivation would be associated with differences in referrals, and controlling for socioeconomic deprivation would reduce the between-neighbourhood variance in family referrals to non-significance. A high level of socioeconomic deprivation was found to be negatively associated with family referrals, indicating that children living in neighbourhoods characterised by high levels of socioeconomic deprivation are less likely to be referred to the child and adolescent psychiatric clinics by their families. However, further research is needed to identify the mechanism explaining how socioeconomic deprivation affect families help seeking behaviour.

The absence of neighbourhood effects on the other referral sources indicates that it is individual characteristics in the child or adolescent rather than where the child/adolescent live that influences referrals by social/legal agencies, schools, or health/mental health care services.

### Limitations

A number of limitations associated with the study should also be mentioned. First, there is no information available on who first initiated the contact with child and adolescent psychiatric clinics. This is an area that requires further research, since it is possible that children who are initially referred by their families are younger and that their problems therefore have been recognised at an earlier stage. It is also possible that most re-referrals are family/self-referrals as a consequence of families having greater knowledge of the care system after the initial contact with CAP-clinics. Though, the analysis in this study regards comparisons between those children who at any time have been referred by a particular source and those who never have been referred by that same source. Second, the proportion of children with an immigrant background is much smaller in this sample than in the population; 18 percent as compared with 28 percent of all the children in the municipality of Stockholm. The underrepresentation of children and adolescents with an immigrant background in the CAP-clinics might be explained in terms of unmet needs, or it may be due to their having fewer mental health related problems than children from the majority population. This is an area that requires further investigation. Family-focused qualitative studies of attitudes towards and knowledge about mental health care for children, and population-based studies of the occurrence of mental health problems are both needed. Third, as a result of data constraints, there is no available information on residential neighbourhood at first appointment. However, figures from Statistics Sweden [48] indicate that (families with) children aged 6-17 do not move frequently and a majority of the moves that do occur are within the same neighbourhood. Fourth, since no information was available on individual/family socioeconomic status, the observed effect of neighbourhood socioeconomic deprivation may just be a compositional effect reflecting individual level socioeconomic status. Previous research has shown that poverty is highly correlated with coercive referrals and it has been found to explain a large part of the observed differences between children from different ethnic backgrounds [21]. However, in Sweden there are no major financial constraints associated with receiving mental health care, and in a review of the literature Zwaanswijk et al. [47] found that in countries with a health system comparable to that of Sweden there was no association between socioeconomic status and help seeking. Finally, neighbourhoods would ideally be operationalised as locally defined areas with natural boundaries [39]. Data of this kind were unfortunately not available for the purposes of this study and neighbourhoods were therefore operationalised as census tracts.

## Conclusions

This article points to the existence of important ethnic and social differences in children's and adolescents' pathways to mental health care. The findings have significant implications for the design and evaluation of community mental health outreach programs, and must be taken into consideration in connection with the development of measures and strategies intended to reach and help children with mental health problems and also to prevent later CAP-care. This might involve providing information about the availability and accessibility of health care for children and adolescents with mental health problems to families in certain areas and to certain ethnic groups. In addition, professionals working with children and adolescents, such as child psychiatrists, general practitioners, paediatrics, social services, and school staff, need to be aware of the differences in referral patterns between children from different neighbourhoods and with different ethnic backgrounds.

## Competing interests

The authors declare that they have no competing interests.

## Authors' contributions

A-KI designed the study, performed the statistical analysis and wrote the manuscript with support from the other co-authors. RS helped with the interpretation of the statistical analysis, revised the manuscript and contributed with valuable comments on the content.

HA, SL, P-AR, and MTL were responsible for designing the database and collecting the data. They have also revised the manuscript and contributed with valuable comments on the content. MTL has also supervised the writing process. All authors have read and approved the final manuscript.

## Appendix

In order to not lose too many level two units, we choose to use only 10 individuals per higher level unit in this study. The disparity in the number of children per neighbourhood is adequately dealt with by the multilevel regression analysis [45]. The general recommendation is to have at least 25 individuals per level two unit, however some studies have shown that if the number of neighbourhoods is large enough, the impact of having only a few individuals per unit is rather small [49].
